# Serum DKK-1 level in ankylosing spondylitis: insights from meta-analysis and Mendelian randomization

**DOI:** 10.3389/fimmu.2023.1193357

**Published:** 2023-07-12

**Authors:** Xi Fang, Cong Chen, Zhi-Xin Wang, Yan Zhao, Ling-Qiong Jiang, Yang Fang, Ruo-Di Zhang, Hai-Feng Pan, Sha-Sha Tao

**Affiliations:** ^1^ Department of Epidemiology and Biostatistics, School of Public Health, Anhui Medical University, Hefei, Anhui, China; ^2^ Institute of Kidney Disease, Inflammation and Immunity Mediated Diseases, The Second Hospital of Anhui Medical University, Hefei, Anhui, China; ^3^ Department of Rheumatology and Immunology, The First Affiliated Hospital of Anhui Medical University, Hefei, Anhui, China

**Keywords:** Dickkopf-1, ankylosing spondylitis, serum level, meta-analysis, Mendelian randomization analysis

## Abstract

**Objective:**

The purpose of this study was to precisely evaluate the serum Dickkopf-1 (DKK-1) level in patients with ankylosing spondylitis (AS) relative to that in normal controls and to test the causal relationship between DKK-1 and the risk of AS.

**Methods:**

Embase, PubMed, Web of Science, WANFANG DATA, VIP, and China National Knowledge Infrastructure (CNKI) were comprehensively searched until July 2022 for pertinent studies. The pooled standardized mean difference (SMD) with a 95% confidence interval (CI) was calculated by the fixed or random-effect model. In Mendelian randomization (MR) analysis on the causal relationship between serum DKK-1 level and AS risk, the inverse variance weighting method (IVW), MR-Egger regression, weighted median method, and weighted pattern method were applied. Sensitivity analyses, including the horizontal pleiotropy test, heterogeneity test, and leave-one-out test, were also performed.

**Results:**

The meta-analysis of 40 studies containing 2,371 AS patients and 1,633 healthy controls showed that there was no significant difference in DKK-1 serum level between AS patients and normal controls (pooled SMD=0.207, 95% *CI* =−0.418-0.832, *P=*0.516). The subgroup analysis of the CRP ≤ 10 mg/L group showed that AS patients had higher serum DKK-1 concentration than the healthy controls (SMD=2.267, 95% *CI* = 0.102-4.432, *P*=0.040). Similarly, MR analysis also demonstrated no significant association between DKK-1 serum level and AS (IVW *OR*=0.999, 95% *CI* = 0.989-1.008, *P=*0.800). All sensitivity analyses revealed consistent results.

**Conclusions:**

There was no significant change in serum DKK-1 concentration between AS patients and healthy controls. In addition, no causal relationship exists between serum DKK-1 levels and AS risk.

## 1 Introduction

Ankylosing spondylitis (AS) is a type of spondyloarthropathy (SpA), which is immune-mediated and associated with a chronic inflammatory response. The primary pathological changes of AS are spinal and sacroiliac joint involvement, excessive ossification and inflammatory osteopenia, spinal joint fusion, bamboo-like shape, reduced mobility resulting in spinal rigidity and back pain, and other clinical manifestations (1). The worldwide prevalence of AS is 0.07%-0.32%, and men are more susceptible to the disease than women (2). It not only places a burden of disease on patients but also increases the social burden due to loss of quality of life and psychological harm to patients (3). Early diagnosis and biological therapy can enhance the life quality of patients to some extent by decreasing the incidence of spinal joint fusion (4). The etiology of AS has not been elucidated. Several relevant factors were revealed to be associated with the risk of AS including genetic, immune, microbial, and endocrine factors (1). In terms of immune factors, pro-inflammatory cytokines, such as tumor necrosis factor-α (TNF-α) and interleukin 17 (IL-17), have been known to play important roles in the etiology of AS (5). Dickkopf-1 (DKK-1) can interact with these cytokines and it may participate in inflammation and autoimmune responses (6). The Wnt signaling pathway is a crucial pathway in the new bone formation mechanism of AS, and the DKK-1 protein on the pathway may affect the occurrence of AS.

As the founding member of the DKK family, DKK-1 is a cysteine-rich secretory glycoprotein that strongly inhibits the Wnt/β-catenin pathway and is soluble (7). DKK-1 competes with Wnt ligand, occupies low-density lipoprotein receptor-associated protein 5/6 (LRP5/6), and binds frizzled proteins to promote the internalization of LRP5/6 receptor, thus inhibiting downstream Wnt signaling, further leading to the degradation of β-catenin and limiting the expression of Wnt target genes (8, 9). One previous study has shown that the serum level of DKK-1 bounding to LRP6 is down-regulated in AS patients (10). The obstruction of this typical Wnt pathway by DKK-1 may interfere with bone metabolism by affecting osteoblasts and osteoclasts (11, 12).

Many studies have been conducted to investigate the serum DKK-1 level of AS patients. A previous meta-analysis including 7 case-control studies from 2010 to 2012 indicated that the level of serum DKK-1 in patients with AS was significantly higher than that in healthy controls (13). However, another recent meta-analysis containing 23 studies from 2010-2017 revealed no significant difference in serum DKK-1 concentration between AS patients and healthy controls (14). Furthermore, more recent studies have reported controversial results since 2018 (15–30). The reason for the inconsistent results may be due to the potential bias of traditional epidemiology, such as measurement error, reverse causality, confounding bias, different races, and clinical heterogeneity, which are caused by the nature of the study design (31). Therefore, whether serum DKK-1 level is causally linked to AS remains undetermined.

In the present study, a two-stage design was applied. First, to derive a more precise estimation of the serum level of DKK-1 in AS patients, a meta-analysis was performed. Second, to determine the causal relationship between serum DKK-1 level and the risk of AS, a Mendelian randomization (MR) analysis was conducted.

## 2 Materials and methods

### 2.1 Data sources

This meta-analysis was performed according to the Preferred Reporting Items for Systematic Reviews and Meta-Analyses (PRISMA) guidelines published in 2021. We systematically conducted an electronic literature retrieval to search all available studies published up to July 2022 that were related to the correlation between DKK-1 serum levels and AS. The online searchable literature libraries included Embase, PubMed, Web of Science, WANFANG DATA, VIP, and the China National Knowledge Infrastructure (CNKI). Our search terms included ankylosing spondylitis, AS, DKK-1, Dickkopf-1, and Dickkopf-Related Protein 1, using subject terms and keywords. Inclusion criteria for the study were as follows: (1) the study was an observational study; (2) cases were patients with AS diagnosed by clear diagnostic criteria, and controls were healthy participants; (3) the study provided data on serum DKK-1 levels in AS patients and controls, such as mean and standard deviation (mean ± SD) or mean and standard error of the mean (mean ± SEM) or median and interquartile range (median (IQR)); (4) the article was written in English or Chinese. Exclusion criteria for the study were as follows: (1) the study was a conference abstract, review, meta-analysis, etc.; (2) the study had no sufficient data; (3) the study was based on animal models or only PBMCs; (4) the study used duplicate data. Moreover, references to the included literature and related reviews were searched to supplement any relevant studies.

Single-nucleotide polymorphisms (SNPs) associated with DKK-1 were screened from genome-wide association studies (GWAS) with 21,758 individuals as instrumental variables (IV), including 13, 102 756 variant loci (32). SNPs associated with the risk of AS were extracted from a European population study, which included 1,476 AS cases and 386,233 controls. The GWAS was derived from the exome sequencing data of the UK Biobank (UKB) Exome Sequencing Consortium, an organization that concentrates on each protein-coding gene in the genome by examining the exome sequences of 454,787 UKB participants (33). All data were downloaded from the publicly available GWAS Catalog database at https://www.ebi.ac.uk.

### 2.2 Data extraction and quality assessment

Two investigators extracted the study information, including the name of the first author, publication year, region of research, individual characteristics (age, gender), serum DKK-1 levels [mean ± SD, mean ± SEM, or median (IQR)], measurement method, duration of disease, and so on, from each of the included studies independently. For any study where there was disagreement regarding the inclusion or extraction of its data, our solution was to discuss it with the third reviewer until a consensus was reached. Two investigators evaluated the methodological quality of each study by the Newcastle-Ottawa scale (NOS) independently. The assessment scale defined articles with scores above 7 as high.

### 2.3 Instrument selection

Quality assessment of DKK-1 data was performed to incorporate optimal IV according to the following requirements. First of all, SNPs significantly correlated with DKK-1 were selected according to the threshold of the *P* value. A set of SNPs with genome-wide statistical significance (*P* < 5×10^-8^) were extracted as IVs. Second, SNPs with minor allele frequencies (MAF) less than 0.01 were excluded. Third, linkage disequilibrium (LD) among IVs was eliminated to reduce the bias in results caused by strong LD. We removed strong SNPs by the clumping procedure (*R* ^2^ < 0.001, clumping length = 10 000 kb), and used 1 000 genomes of the European population as a reference. Fourth, corresponding SNPs that affect exposure and outcome should have the same effect alleles, and those with different effector genes should be corrected or deleted to guarantee that palindromic sequences are not included in IVs. Finally, after screening according to the above requirements, if there were no corresponding SNPs that affect exposure and outcome, proxy SNPs should be sought for further analysis (*r^2^* >0.8).

The included IVs should be subject to the following three assumptions: (1) IVs are closely related to exposure; (2) IVs are irrelevant to any confounding factors affecting the exposure-outcome association, F statistic was applied to evaluate the strength of correlation between the two, and those with the F statistic less than 10 were considered to be weakly correlated, which needed to be eliminated; (3) IVs do not affect outcomes except by association with exposure. Diabetes and hypertension, as common complications of ankylosing spondylitis, may affect DKK-1 production and function in the body (34–36). In order to exclude their influence as much as possible, we included them as confounding factors for further analysis.

### 2.4 Statistical analysis

R (version 4.1.1) was used for all statistical analyses. The correlation between serum DKK-1 and AS was assessed by calculating the standardized mean difference (SMD) with a 95% confidence interval (CI). Before calculating SMD, the data units were unified, SEM values were converted to SD using the function, SD=SEM× (37), and the median (IQR) references the methods of Zhao et al (38) and Luo et al (39) to convert into mean ± SD. The heterogeneity of the research was examined utilizing Cochran’s Q statistic and I^2^ test (40). The choice of effect model depended on the *P* value and *I^2^*. When *P* < 0.100 or *I^2^* >50%, the random effect model was chosen, and the fixed effect model was chosen for other cases. To further investigate the source of heterogeneity, subgroup analysis and meta-regression were implemented. Sensitivity analysis was carried out by eliminating each study to check the robustness of the results. The assessment of publication bias was adapted through Egger’s linear regression test and Begger’s rank correlation test, and funnel plots were used for its visualization. Outside the heterogeneity test (*P* < 0.100), other tests defined *P <*0.05 as significant.

The MR analysis methods used to estimate the causal effect between exposure and outcome in this study were the inverse variance weighting method (IVW), MR-Egger regression, weighted median method, and weighted method. A sensitivity analysis of the MR analysis results was also needed, which can be divided into three aspects. On one side, Cochran’s Q statistic was applied to test the heterogeneity among SNPs. Second, MR pleiotropy residual sum and outlier (MR-PRESSO) was utilized to test horizontal pleiotropy, eliminate outliers to correct the results, and compare the results before and after correction. Third, a leave-one-out analysis was conducted to examine the robustness and consistency of the result.

## 3 Results

### 3.1 Observed associations between serum DKK-1 and AS

#### 3.1.1 Publication search and study characteristics

**Figure 1:**
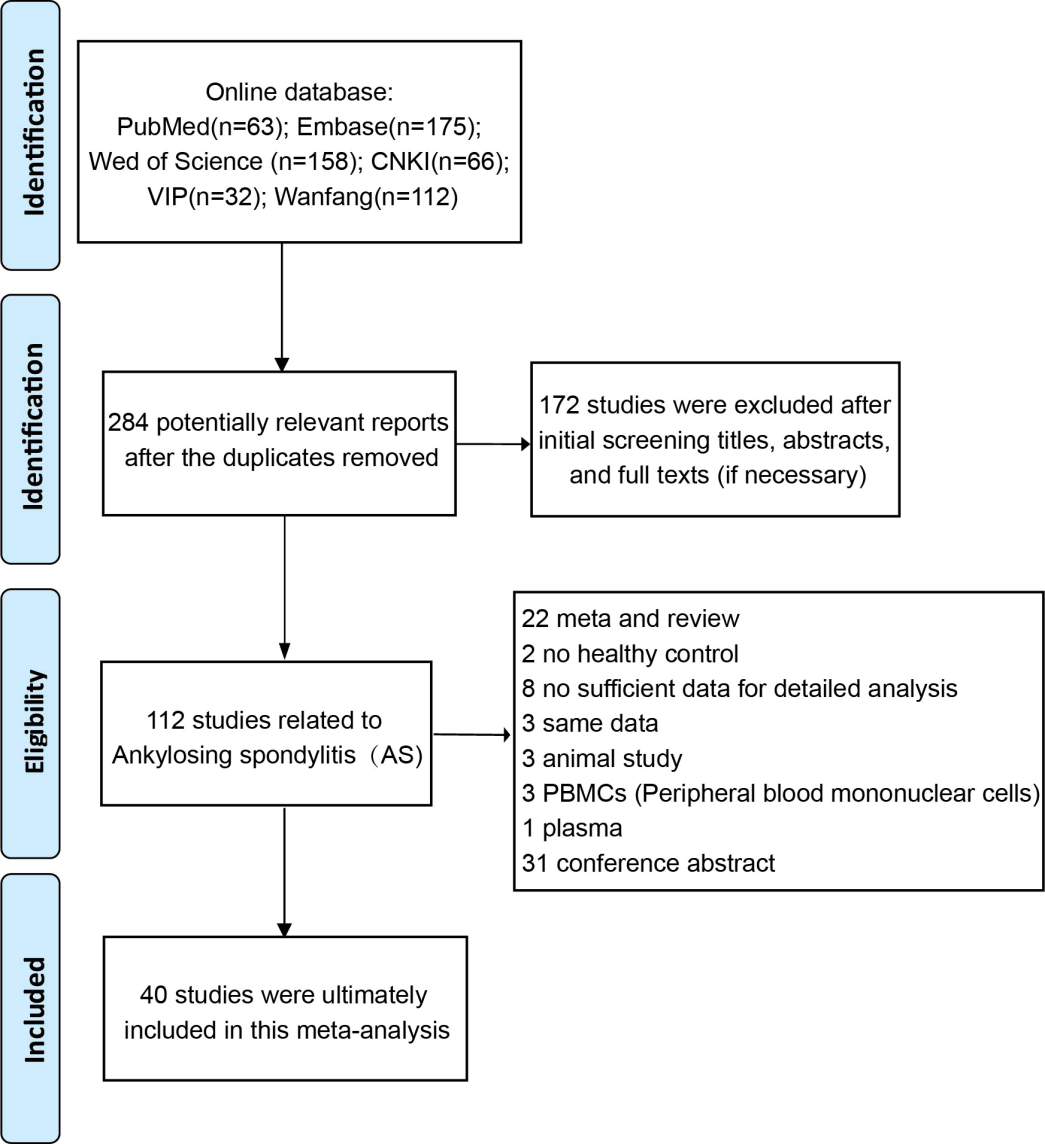
Flowchart of article selection.

The detailed process of literature screening is shown in **Figure 1**. Of the 6,066 articles originally searched, 40 studies published from 2010 to 2022 were ultimately included in the meta-analysis. A total of 2,371 AS patients and 1,633 healthy controls were investigated. Of the 40 studies, 31 were case-control studies and 9 were cross-sectional studies. Furthermore, 6 were conducted in Euramerican and 34 in Asia. All of the studies used enzyme-linked immunosorbent assay (ELISA) to detect the serum levels of DKK-1. All the included articles were of medium to high quality, with NOS scores ranging from 5 to 8. The main information of the literature is shown in **Table 1**.

**Table 1:** General characteristics of included meta-analyses.

Authors	Publication year	Region	Study type	Cases				Controls				*P*	Criteria for the classification of AS	Measurement type
				N	Sex ratio(M/F)	Mean(pg/mL)	SD(pg/mL)	N	Sex ratio(M/F)	Mean(pg/mL)	SD(pg/mL)			
Daoussis D, et al (41)	2010	Greece	Cross-sectional	45	35/10	2730.00	906.28	50	25/25	2375.00	875.40	0.040	ACR	ELISA
Wang SY, et al (42)	2011	China	Case-control	30	NA	4042.90	2283.60	40	7/33	3198.90	2283.60	0.134	NA	ELISA
Shan ZX, et al (43)	2011	China	Case-control	47	39/8	1965.96	120.04	20	NA	1363.00	101.65	<0.05	NA	ELISA
Sui L, et al (44)	2011	China	Case-control	14	7/7	3560.00	830.00	20	10/10	6270.00	1650.00	<0.01	ACR	ELISA
Elshishtawy H, et al (45)	2012	America	Cross-sectional	30	27/3	2539.70	234.90	20	18/2	1635.30	267.40	<0.001	ACR	ELISA
Kim TJ, et al (46)	2012	Korea	Case-control	49	44/5	341.80	385.70	53	47/6	239.60	235.88	>0.05	ACR	ELISA
Kwon SR, et al (47)	2012	Korea	Case-control	56	47/9	12321.00	6136.00	40	31/9	20811.00	5671.00	<0.001	ACR	ELISA
Liu H, et al (48)	2012	China	Case-control	60	NA	20030.00	3590.00	30	NA	15210.00	2600.00	<0.05	NA	ELISA
Korkosz M, et al (49)	2013	Poland	Cross-sectional	50	42/8	818.70	812.47	23	NA	1349.30	396.62	<0.05	ACR	ELISA
Korkosz M, et al (49)	2013	Poland	Cross-sectional	28	NA	1692.70	647.15	23	NA	1349.30	396.62	<0.05	ACR	ELISA
Tuylu T, et al (50)	2014	Turkey	Case-control	45	32/13	1911.00	1344.00	68	48/20	672.00	592.00	<0.001	ACR	ELISA
Tuylu T, et al (50)	2014	Turkey	Case-control	49	33/16	1727.00	1083.00	68	48/20	672.00	592.00	<0.01	ACR	ELISA
Ustun N, et al (51)	2014	Turkey	Cross-sectional	44	34/10	314.96	196.73	41	NA	613.34	861.86	0.062	ACR	ELISA
Zhang YT, et al (52)	2014	China	Case-control	30	25/5	819.65	204.56	20	17/3	1591.50	335.92	<0.05	NA	ELISA
Kong WP, et al (53)	2014	China	Case-control	45	35/15	1625.79	1342.17	40	31/9	2280.97	1216.25	<0.05	ACR	ELISA
Zhou YS, et al (54)	2014	China	Cross-sectional	84	60/24	3627.00	3805.19	79	44/35	3684.00	4605.19	0.276	ACR	ELISA
Zhou YS, et al (54)	2014	China	Cross-sectional	84	60/24	7.24	7.34	79	44/35	9.15	5.94	<0.001	ACR	ELISA
Xie JM, et al (55)	2015	China	Case-control	75	67/8	65.60	23.40	70	61/9	94.70	32.50	<0.01	ACR	ELISA
Xie JM, et al (56)	2015	China	Case-control	55	48/7	72.60	24.50	45	36/9	98.00	31.80	<0.01	ACR	ELISA
Cui YF, et al (57)	2015	China	Case-control	51	40/11	2133.90	432.90	15	NA	1718.70	260.40	0.001	ACR	ELISA
Huang JX, et al (58)	2016	China	Case-control	43	34/8	1914.50	407.80	42	32/10	1729.10	352.90	0.028	ACR	ELISA
Su XJ, et al (59)	2016	China	Case-control	45	36/9	1956.86	55.02	50	39/11	2207.12	95.88	<0.05	ACR	ELISA
Rossini M, et al (60)	2016	Italy	Cross-sectional	71	59/12	668.24	375.71	71	NA	854.66	456.01	0.009	ACR	ELISA
Sakellariou GT, et al (61)	2017	Greece	Cross-sectional	57	53/4	1629.50	928.63	34	32/2	1388.80	780.76	0.111	ACR	ELISA
Niu CC, et al (62)	2017	China	Case-control	6	5/1	379.80	48.10	9	2/7	792.50	308.60	<0.01	NA	ELISA
Bai J, et al (63)	2017	China	Case-control	46	30/16	720.00	150.00	38	28/10	120.00	30.00	<0.05	ACR	ELISA
Park JH, et al (64)	2017	Korea	Case-control	20	NA	695.97	260.00	11	NA	565.75	287.41	0.087	ACR	ELISA
Jadon DR, et al (65)	2017	Britain	Cross-sectional	157	118/39	3520.00	1288.89	50	26/24	3510.00	1229.63	0.080	ACR	ELISA
Sohn DH, et al (18)	2018	Korea	Cross-sectional	55	NA	2052.10	1153.93	26	NA	1358.70	715.41	0.039	ACR	ELISA
Solmaz D,et al (19)	2018	Turkey	Case-control	97	76/21	368.00	123.70	48	36/12	367.00	547.41	0.970	ACR	ELISA
Liao HT, et al (17)	2018	China	Case-control	72	58/14	332.18	138.44	30	NA	643.84	162.02	0.003	ACR	ELISA
Zhang Z, et al (16)	2018	China	Case-control	46	30/16	710.00	160.00	38	28/10	130.00	40.00	<0.01	ACR	ELISA
Sun J, et al (15)	2018	China	Case-control	37	22/15	2135.60	445.20	74	38/36	1711.50	260.40	<0.05	NA	ELISA
Sun W, et al (23)	2019	China	Case-control	88	66/22	1855.00	84.58	26	NA	1406.00	99.76	<0.05	ACR	ELISA
Wang TL, et al (20)	2019	China	Case-control	40	36/4	1162.00	390.00	20	NA	2237.00	370.00	<0.01	NA	ELISA
Liu DD, et al (21)	2019	China	Case-control	40	28/12	1663.90	182.00	40	25/15	1310.40	121.70	<0.001	ACR	ELISA
Jiao AJ, et al (22)	2019	China	Case-control	145	127/18	72.07	7.29	62	51/11	98.19	8.73	<0.001	CMA	ELISA
Liu XL, et al (24)	2020	China	Case-control	40	33/7	606.20	528.70	40	29/11	991.80	814.10	0.014	ACR	ELISA
Pei F, et al (25)	2020	China	Case-control	110	60/50	1203.37	148.20	100	56/44	992.73	115.26	<0.05	CMA	ELISA
Wang C, et al (26)	2020	China	Case-control	60	49/11	3670.00	940.00	60	45/11	6380.00	1760.00	<0.001	CMA	ELISA
Hu JL, et al (27)	2021	China	Case-control	40	34/6	70. 88	8.34	40	32/8	81.03	9.81	<0.01	ACR	ELISA
Hu JL, et al (28)	2021	China	Case-control	50	35/15	70.28	7.92	50	33/17	80.87	9.83	<0.001	ACR	ELISA
Jo SS, et al (29)	2021	Korea	Case-control	103	NA	917.40	514.59	30	NA	826.20	190.74	0.043	ACR	ELISA

ACR, American College of Rheumatology; CMA, Chinese Medical Association; NA, not available.

#### 3.1.2 Overall effects

**Figure 2:**
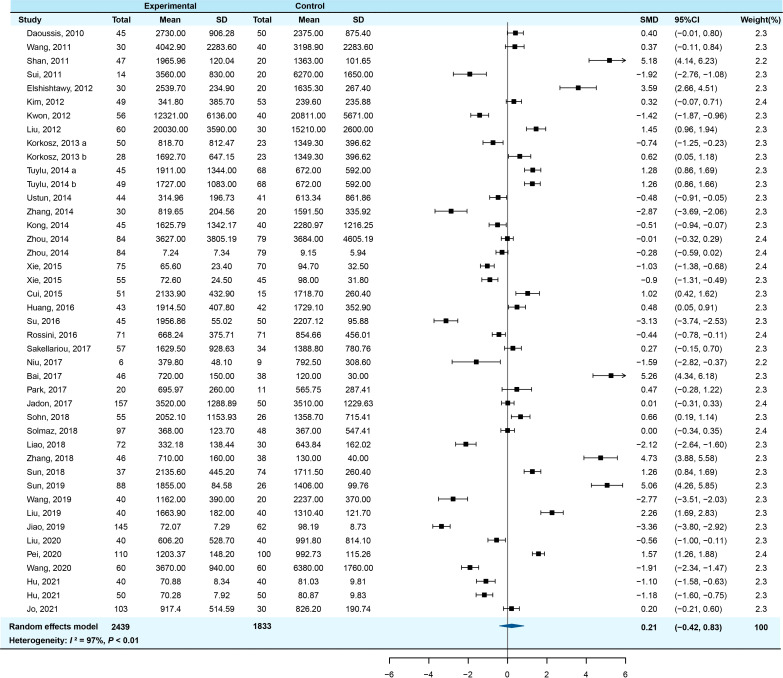
Forest plot of serum DKK-1 levels for AS patients vs healthy controls (a/b means the different subgroup from the same research).

Meta-analysis of the overall articles showed significant heterogeneity (*I* ^2 ^= 97.5%, *P*<0.001), therefore the random-effect model was used to calculate the effect sizes. The serum DKK-1 levels of AS patients had no significant change compared with controls (pooled SMD=0.207, 95% *CI* = −0.418-0.832, *P=*0.516). The forest plot of SMDs (95% CI) of serum DKK-1 in AS patients relative to controls is shown in **Figure 2**.

#### 3.1.3 Subgroup analysis and meta-regression analysis

**Figure 3:**
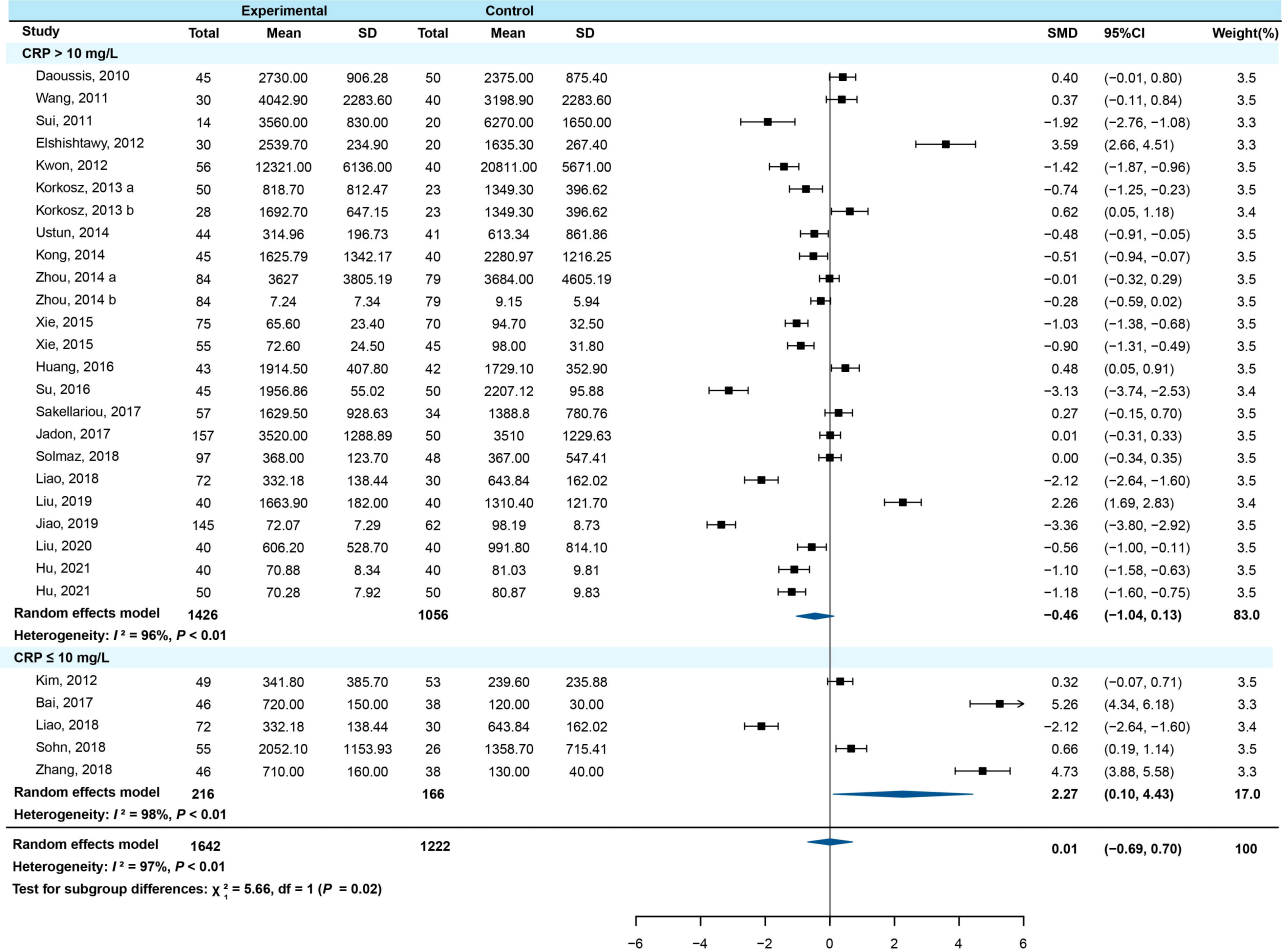
Forest plot with subgroup analysis of CRP (a/b means the different subgroup from the same research).

Subgroup analysis was conducted by stratification according to study type, age, region, course of disease, C-reactive protein (CRP), erythrocyte sedimentation rate (ESR), the Bath AS Disease Activity Index (BASDAI), and the modified Stroke AS Spine Score (mSASSS). The results showed that among the CRP ≤10 mg/L subgroup, the serum DKK-1 concentration in AS patients was higher than that in healthy controls (SMD=2.267, 95% *CI* = 0.102-4.432, *P*=0.040) (**Figure 3**). The other subgroups all had no statistical significance (all *P* > 0.05) (**Supplementary Figures 1–7**).

The results of meta-regression analysis on NOS, publication year, and research sample size showed that none of them were potential sources of heterogeneity (all *P >*0.05) (**Table 2**).

**Table 2:** Meta-regression analysis for the basic characteristics of the study.

Variables	beta	se	95% CI	P value
Sample size	-0.0004	0.0098	(-0.0195, 0.0187)	0.967
NOS	-0.4186	0.4702	(-1.3401, 0.5030)	0.373
Publication year	-0.0565	0.1280	(-0.3074, 0.1945)	0.659

#### 3.1.4 Publication bias and sensitivity analysis

**Figure 4:**
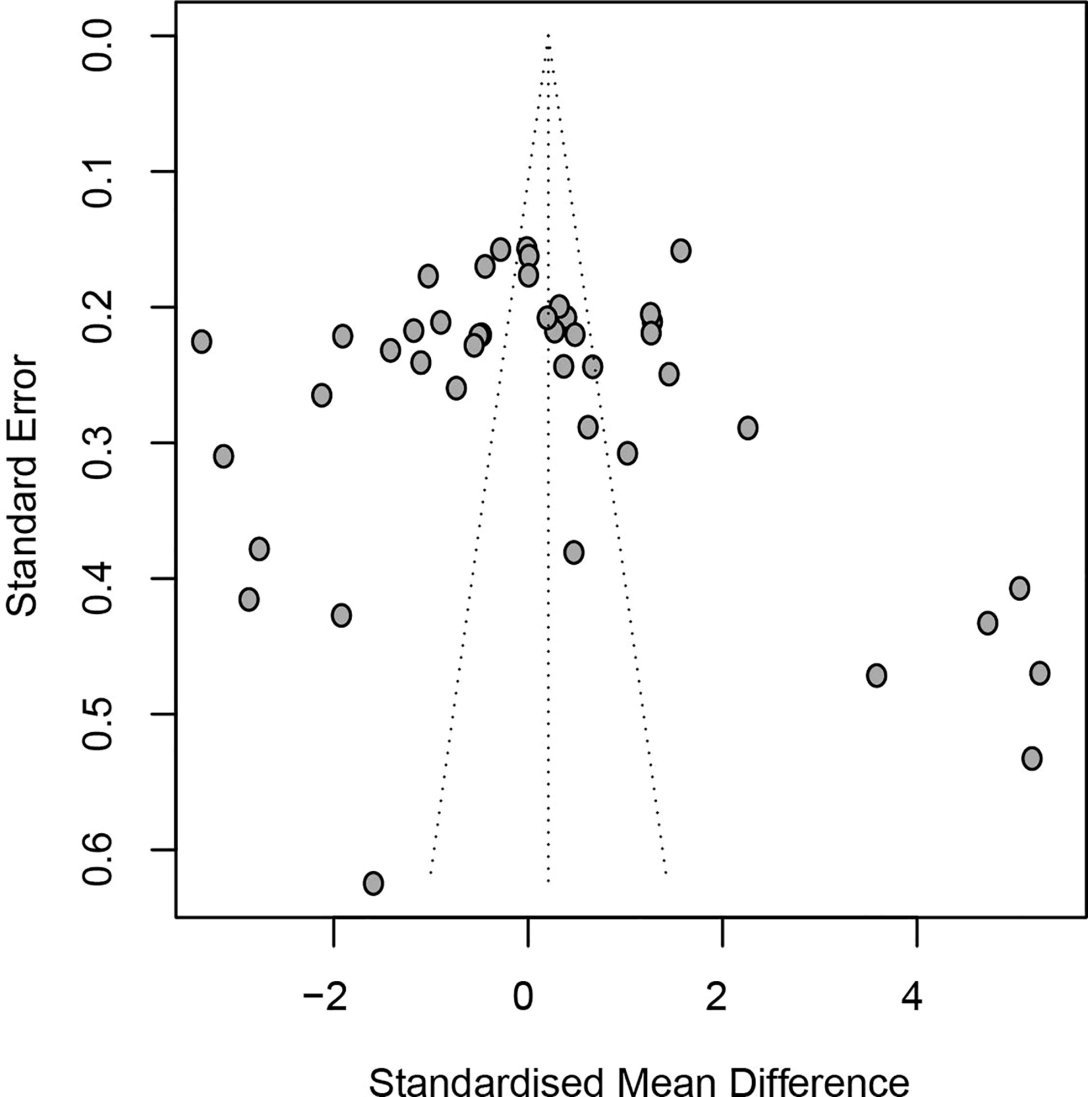
Funnel plots assessing publication bias of included studies.

Egger’s linear regression test (*t* = 1.14, *P* = 0.260) and Begger’s rank test (*Z* = 0.58, *P* = 0.565) did not detect any publication bias (**Figure 4**). A sensitivity analysis of all the included studies indicated that none of the 40 studies changed the overall effect size (all *P >*0.05), suggesting the results were robust.

### 3.2 Causal associations between serum DKK-1 and AS

#### 3.2.1 IV selection

Initially, 579 were extracted as IVs from a large-scale GWAS. After eliminating SNPs that had LD effects, 9 SNPs were selected as IVs. The calculated F-statistic was 76.0, greater than 10, so there was no weak IV in these 9 SNPs.

#### 3.2.2 Two-sample MR analysis

**Figure 5:**
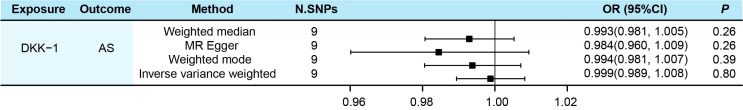
Evaluated causal relationships between DKK-1 and AS using different MR methods.

The result of IVW showed no evidence of an association between serum DKK-1 levels and the risk of AS (IVW *OR*= 0.999, 95% *CI* = 0.989-1.008, *P =* 0.800) (**Figure 5**). MR-Egger regression was used to assess the horizontal pleiotropy among IVs and outcome, and the results showed that there was no horizontal pleiotropy between them (*P* = 0.261). For the sensitivity analysis, no outliers were found in the analysis by MR-PRESSO (*P* = 0.692). The results of Cochran’s Q test indicated that there was no significant heterogeneity (Q= 5.527, *P* = 0.700) in the effect of DKK-1-related SNPs on the risk of AS, which revealed the reliability of MR results. Through the leave-one-out test of eliminating each SNP, the consistency of the results indicated the stability of the MR analysis results (**Supplementary Table 2**). There was no statistical significance between DKK-1 and confounding factors (All *P* > 0.05) (**Supplementary Tables 3, 4**).

## 4 Discussion

Ectopic formation of new bone and fusion of spinal joints in axial bones are vital factors in the disease burden of AS, and the β-catenin-dependent canonical Wnt signaling pathway has been investigated and proved to be an essential mechanism for osteogenesis and spinal sacralization (66). In a previous observational study exploring the link between Wnt signaling and AS, Diara et al. reported for the first time that the expression level of DKK-1 was lower in AS patients than in controls (10). As a recognized Wnt inhibitor, DKK-1 can be expressed through osteoblasts, antagonize the Wnt pathway, and inhibit osteoblast maturation and new bone formation. Moreover, blocking the Wnt pathway can promote the regulation of osteoclasts (67). DKK-1 can therefore impact osteoblasts and osteoclasts to participate in bone metabolism.

In subsequent studies on the serological level of DKK-1 in patients with AS, some studies (17, 20, 22, 24, 26–28, 44, 47, 49, 51–53, 55, 56, 59, 60, 62) also demonstrated lower serum DKK-1 levels in patients than in controls. In contrast, some studies (15, 16, 18, 21, 23, 25, 43, 45, 48–50, 57, 58, 63) have reported that the serum level of DKK-1 in patients was higher than that in controls. Furthermore, there were also some studies (19, 29, 41, 42, 46, 54, 61, 64, 65) showing that there was no significant diversity in DKK-1 serum levels among AS patients and healthy controls. Our current meta-analysis suggested that there was no significant association between serum DKK-1 and AS. This was similar to the overall effect result in a previous meta-analysis by Wu et al (14). In order to reduce the influence of confounding factors and make causal inference more reliable, we further conducted MR analysis, and the result likewise supported the finding of meta-analysis.

The reasons for the above results can be explained by the following points. First, DKK-1 primarily contributes to the development of new bone during AS (68). However, the expression of DKK-1 is not limited to bone, and it is also highly expressed in T cells, platelets, and a variety of cancer cells, with platelets serving as a significant source of circulating DKK-1 (11). We analyzed some complications as confounding factors, but the influence of other complications could not be excluded. Therefore, it is not accurate to ascribe DKK-1 fluctuation in the human body only to AS. Second, TNF-α and IL-1β can induce DKK-1 and sclerostin (SOST), and IL-6 stimulates B cells to differentiate into DKK-1-expressing plasma cells (6, 7). SOST is also an inhibitor of the Wnt pathway, binding low-density lipoprotein-related receptors 5 and 6 (LRP5/6) to affect Wnt signaling, and when DKK-1 is blocked, SOST may produce a compensatory response to restore Wnt signaling to a stable state (69). DKK-1 may interact with the molecules listed above to regulate Wnt signaling, which affects bone metabolism. Third, functional DKK-1 can validly play its role in blocking the Wnt pathway; however, when it is dysfunctional, it may not bind tightly to LPR5/6, resulting in the inability to inhibit Wnt signaling (34, 70). A previous study has shown that anti-TNF therapy can effectively inhibit the inflammatory response in the treatment of AS, but cannot prevent bone fusion, which may be related to DKK-1 disability (71). In addition, non-steroid anti-inflammatory drugs (NSAIDs) also affect the expression level of DKK-1 in patients, and different treatment regimens may lead to clinical heterogeneity (17).

The subgroup analysis results of the normal CRP group (CRP ≤ 10 mg/L) showed that AS patients had higher serum DKK-1 levels relative to the healthy controls. This result was partially identical to the results reported by Wu et al (14). The above phenomenon may also be related to the relative balance of proinflammatory cytokines, Wnt pathway inhibitors, and DKK-1 in the human body environment. The different results between the CRP subgroup and the ESR subgroup are probably due to the different rates at which the two indicators reflect inflammation in the body. CRP and ESR are indicators of the inflammatory response in the acute phase. CRP has higher sensitivity and can reflect the inflammatory state of the body more timely and accurately, and CRP is currently the most sensitive biomarker reflecting the disease activity of AS (72, 73). Therefore, it is not clear whether DKK-1 can reflect the inflammation of AS.

There were some limitations in our study. First, in the meta-analysis, there was significant heterogeneity in the results. Despite our subgroup and meta-regression analyses, the source of heterogeneity was still unknown. Second, the accuracy of reagents and instruments used to detect DKK-1 levels in various studies was not uniform, which was crucial to the research results. Third, the effect of participants’ medications on DKK-1 fluctuations in the body was uncertain, potentially reversing the findings. Fourth, in the MR analysis stage, the data were all from the European population, thus the extrapolation of the MR analysis results to other populations of different races should be treated with caution. Our study also had several strengths. First, the meta-analysis summarized all existing studies related to this study, increasing the sample size of the data and improving the accuracy of the results. Second, MR analysis used genetic polymorphisms of genes to select IVs from GWAS to explore the relationship between exposure and outcome (74). This can minimize confounding bias and avoid reverse causality because genetic variation occurs before disease, which makes the result more reliable (75). Third, some confounding factors that may affect the results of the MR analysis were analyzed to further reduce confounding bias. Finally, both meta-analysis and MR analysis showed that there was no statistical correlation between serum DKK-1 level and AS, so the results were more realistic and reliable. Based on these results, it can be known that no difference in the expression level of DKK-1 between AS patients and controls has been found at the serum level and gene level, and it is uncertain whether DKK-1 can be used as a clinical indicator of AS.

## 5 Conclusion

The results of the meta-analysis and MR analysis both revealed that there was no significant association between serum DKK-1 concentration and AS. The molecular mechanism of DKK-1 and other cytokines in the occurrence of AS still warrants further study.

## Data availability statement

The original contributions presented in the study are included in the article/**Supplementary Material**. Further inquiries can be directed to the corresponding authors.

## Author contributions

S-ST and H-FP conceived the idea and proofread the manuscript. XF drafted and revised the manuscript. CC participated in formal analysis and validation. Z-XW and YZ participated in literature search and information collection. L-QJ and YF participated in visualization. R-DZ participated in language editing. All authors contributed to the article and approved the submitted version.

## Glossary

**Table:**

DKK-1	Dickkopf-1
AS	Ankylosing spondylitis
CNKI	China National Knowledge Infrastructure
SMD	Standardized mean difference
CI	Confidence interval
MR	Mendelian randomization
IVW	Inverse variance weighting method
SpA	Spondyloarthropathy
TNF-α	Tumor necrosis factor-α
IL-17	Interleukin 17
PRISMA	Preferred Reporting Items for Systematic reviews and Meta-Analyses
SD	Standard deviation
SEM	Standard error of the mean
IQR	Interquartile range
SNP	Single-nucleotide polymorphism
GWAS	Genome-wide association studies
IV	Instrumental variable
UKB	UK Biobank
NOS	Newcastle-Ottawa scale
MAF	Minor allele frequencies
LD	Linkage disequilibrium
MR-PRESSO	Mendelian randomization pleiotropy residual sum and outlier
ELISA	Enzyme-linked immunosorbent assay
CRP	C-reactive protein
ESR	Erythrocyte sedimentation rate
BASDAI	The Bath AS Disease Activity Index
mSASSS	The modified Stroke AS Spine Score
SOST	Sclerostin
NSAIDs	Non-steroid anti-inflammatory drugs
LRP5/6	Low-density lipoprotein-related receptors 5 and 6.
