# Supplementary material for: Serum DKK-1 level in ankylosing spondylitis: insights from meta-analysis and Mendelian randomization
Source: Front Immunol. 2023 Jul 12;14:1193357. doi: 10.3389/fimmu.2023.1193357 (PMC10368999; doi:10.3389/fimmu.2023.1193357)

Supplementary Figure 1. Forest plot with subgroup analysis of age


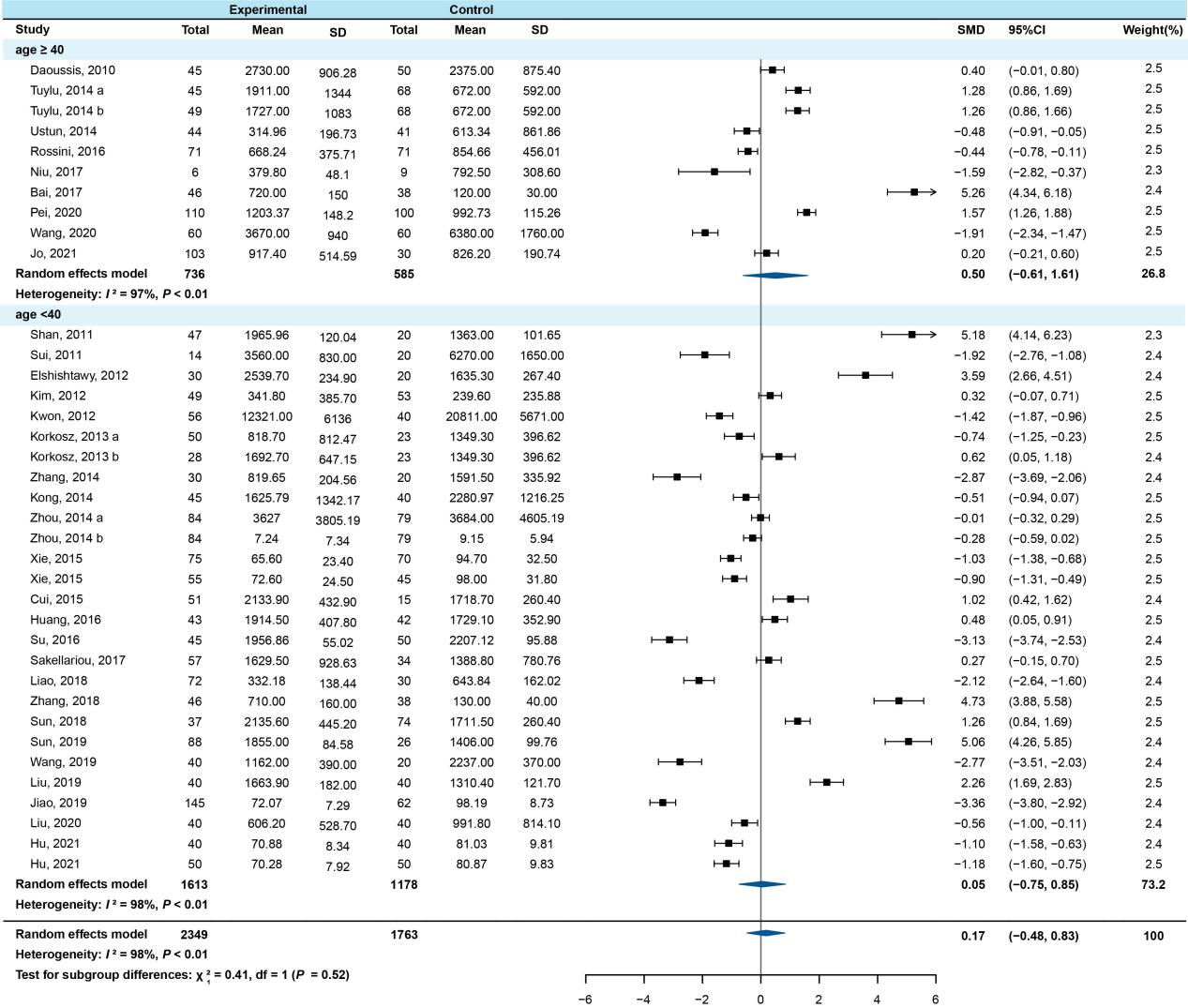


Supplementary Figure 2. Forest plot with subgroup analysis of region


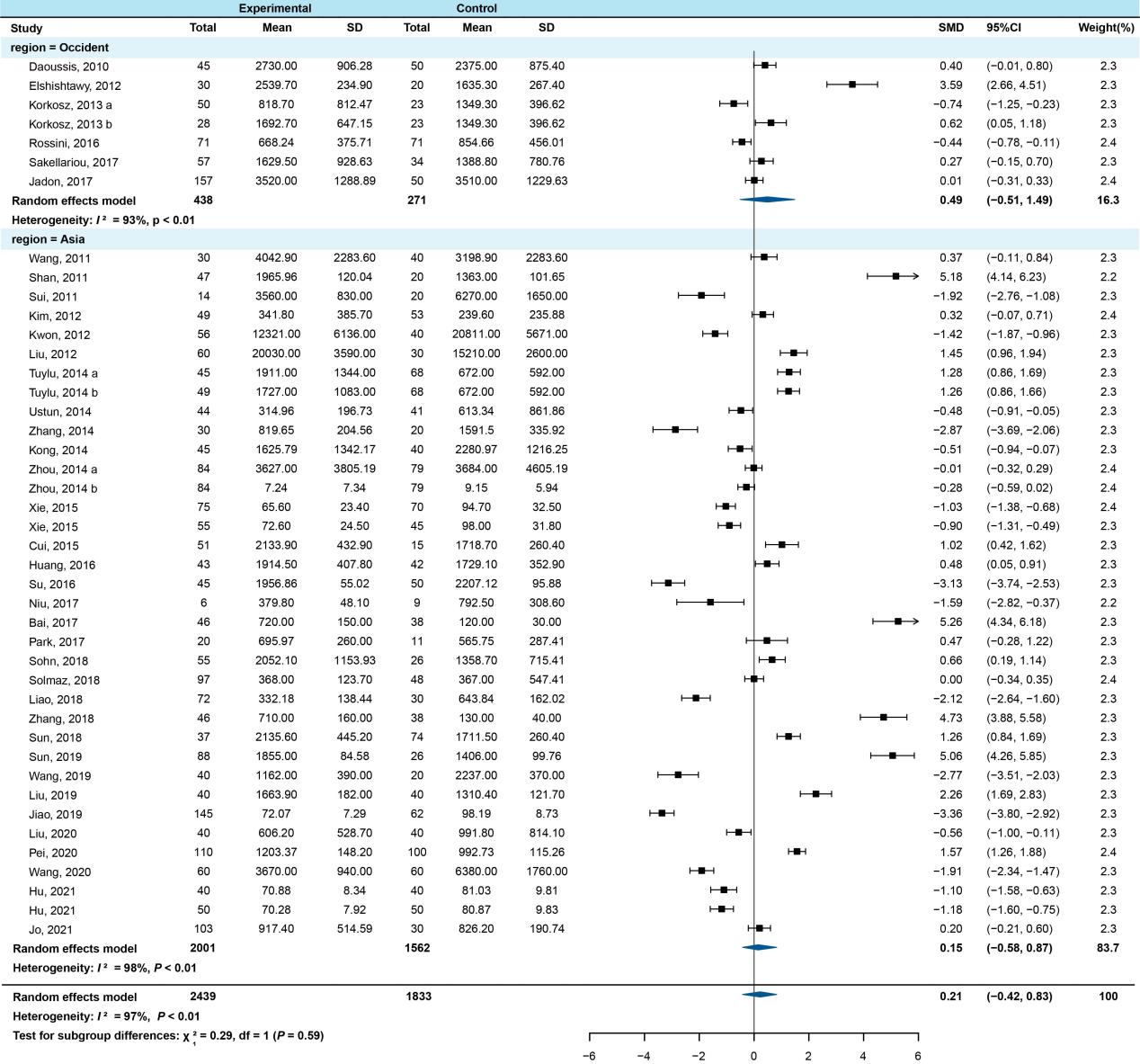


Supplementary Figure 3. Forest plot with subgroup analysis of duration of disease


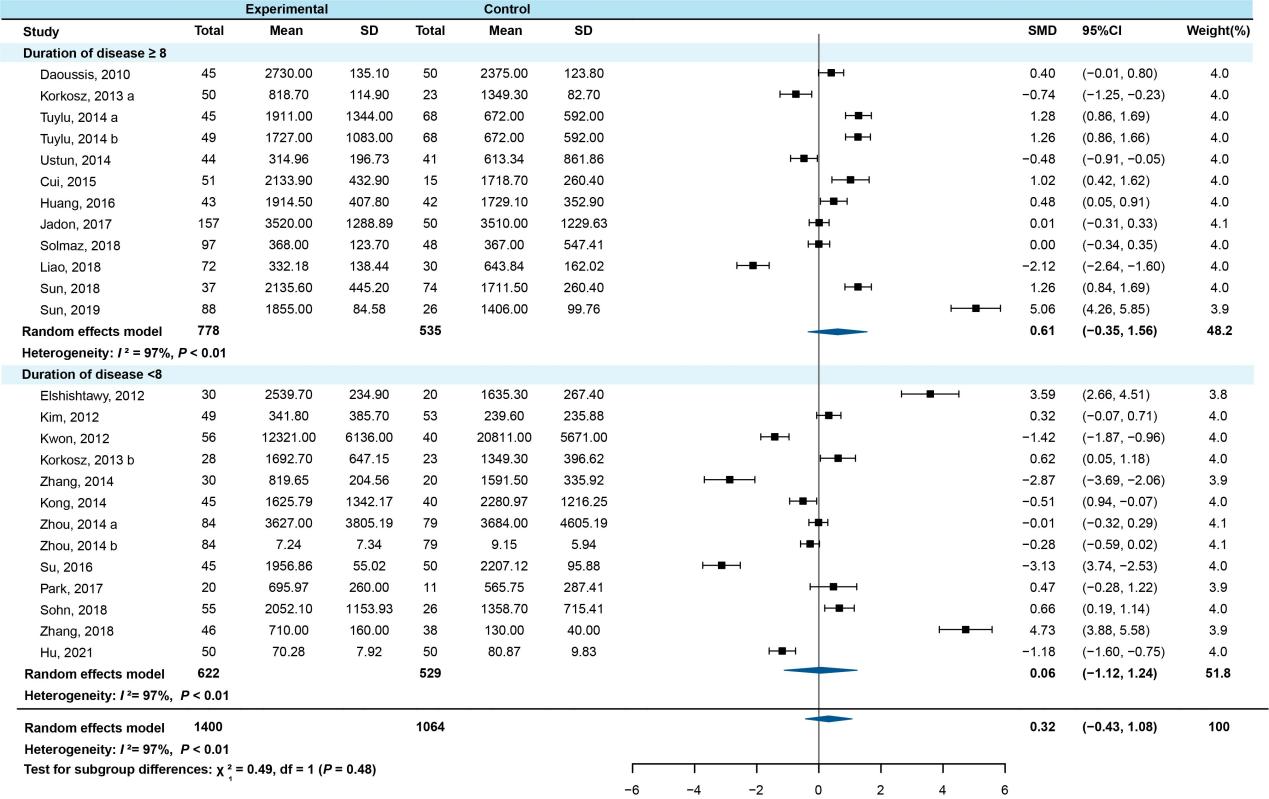


Supplementary Figure 4. Forest plot with subgroup analysis of ESR


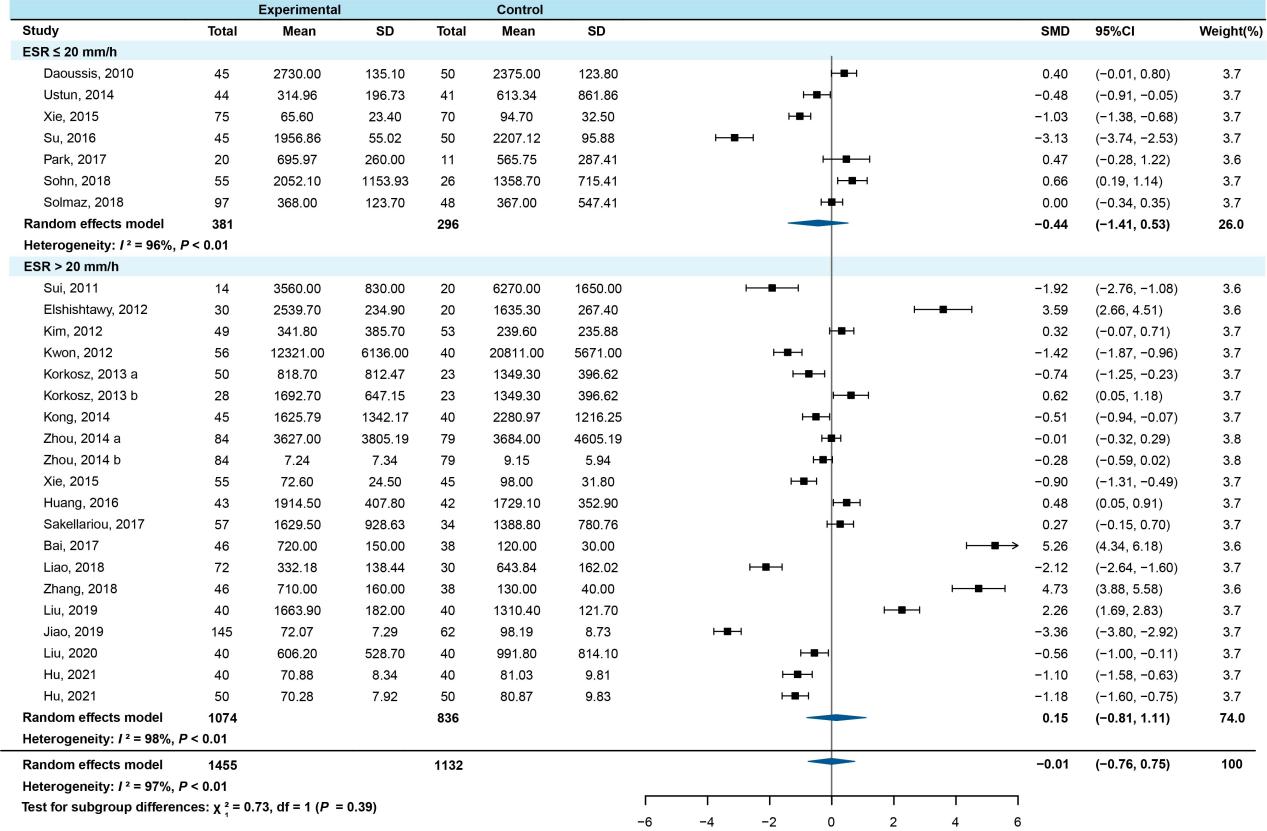


Supplementary Figure 5. Forest plot with subgroup analysis of BASDAI


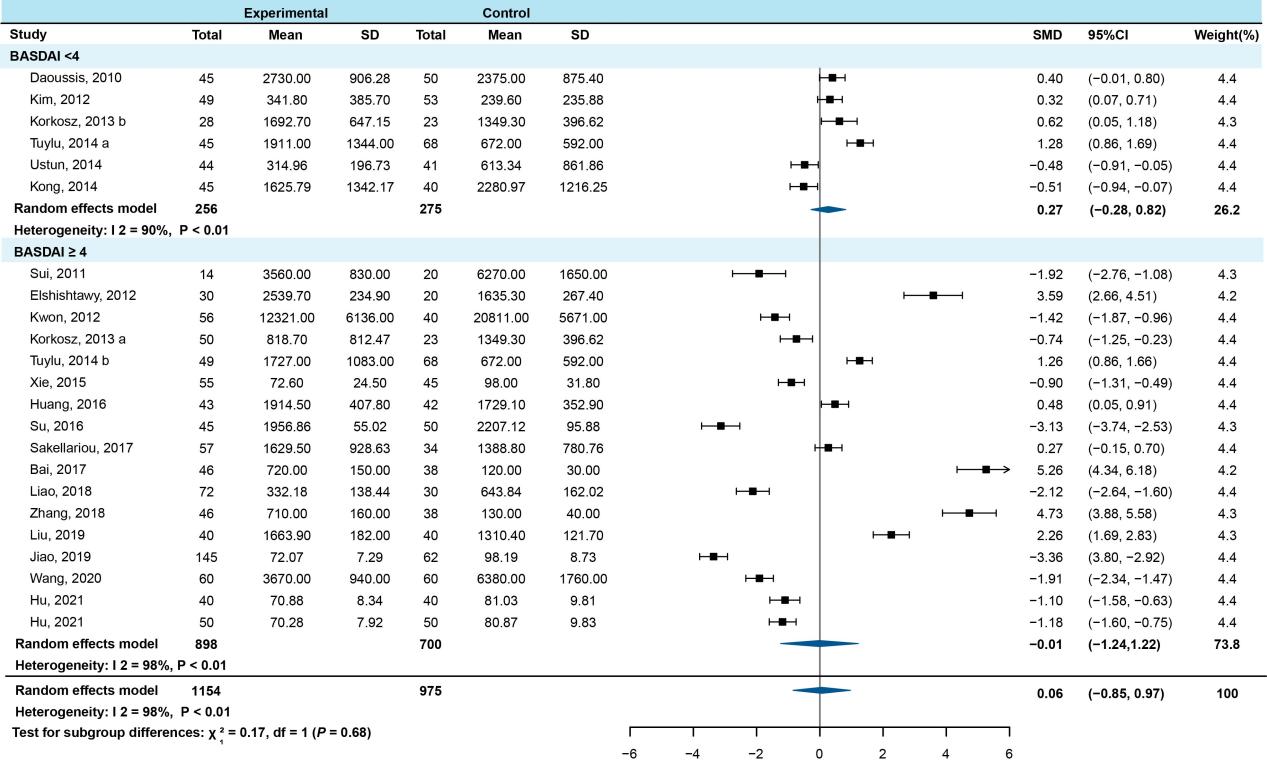


Supplementary Figure 6. Forest plot with subgroup analysis of mSASSS


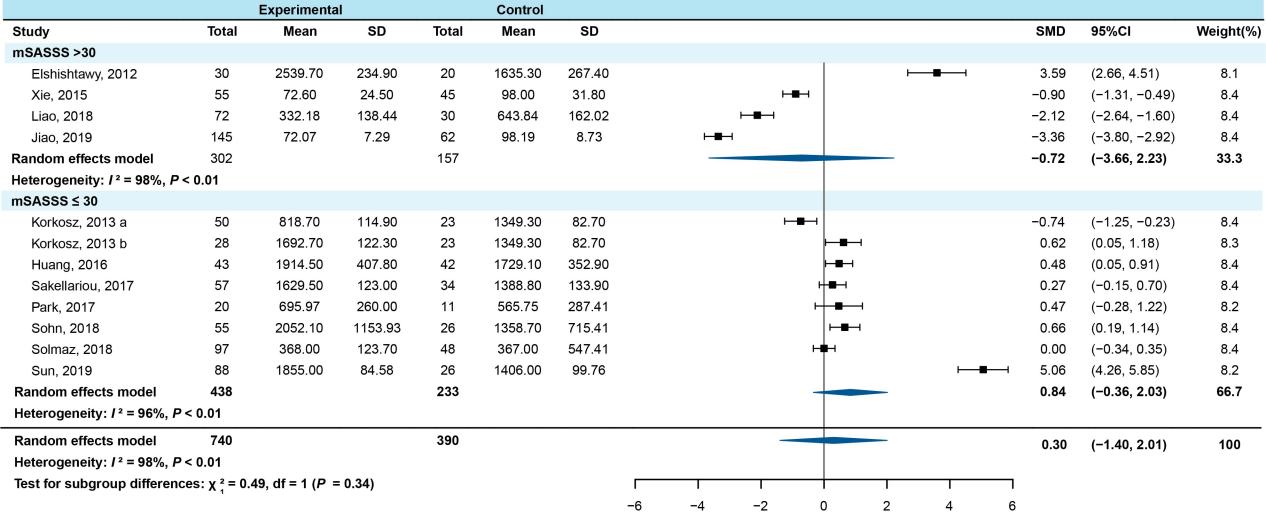


Supplementary Figure 7. Forest plot with subgroup analysis of study type


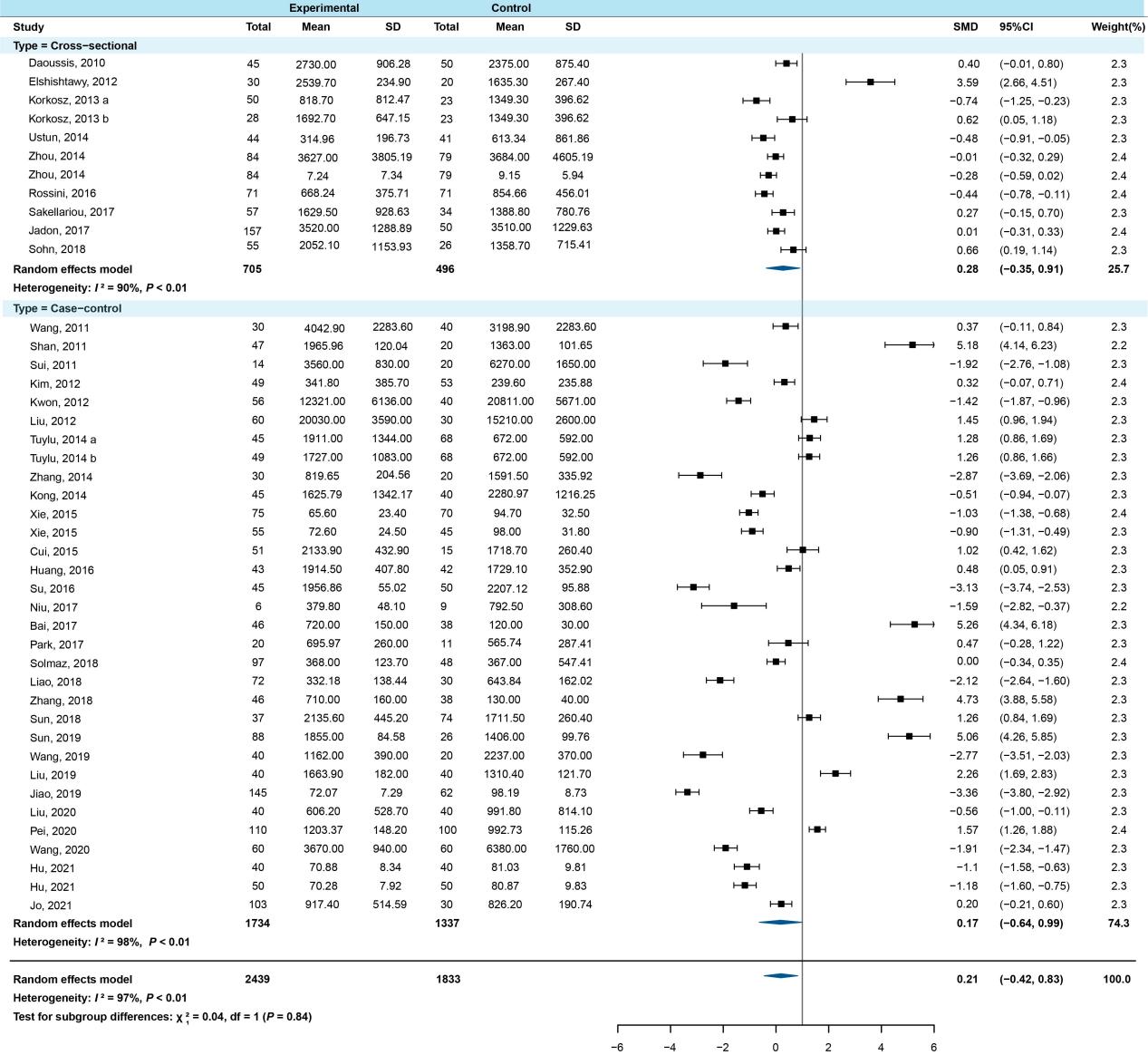


Supplementary Figure 8. Forest plot of sensitivity analysis in meta-analysis


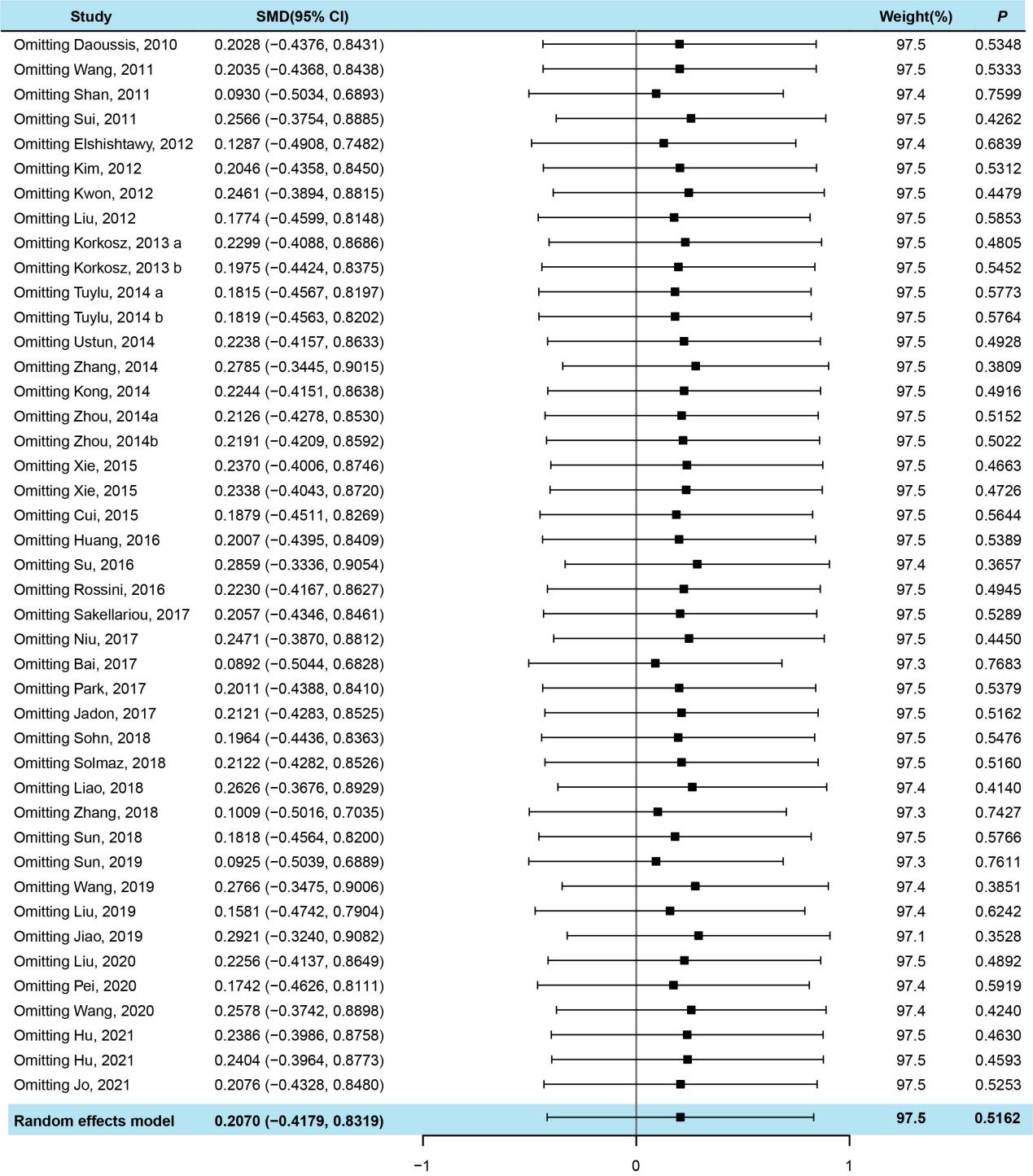


Supplementary Figure 9. Scatter plot of the causality between DKK-1 and AS using different MR methods


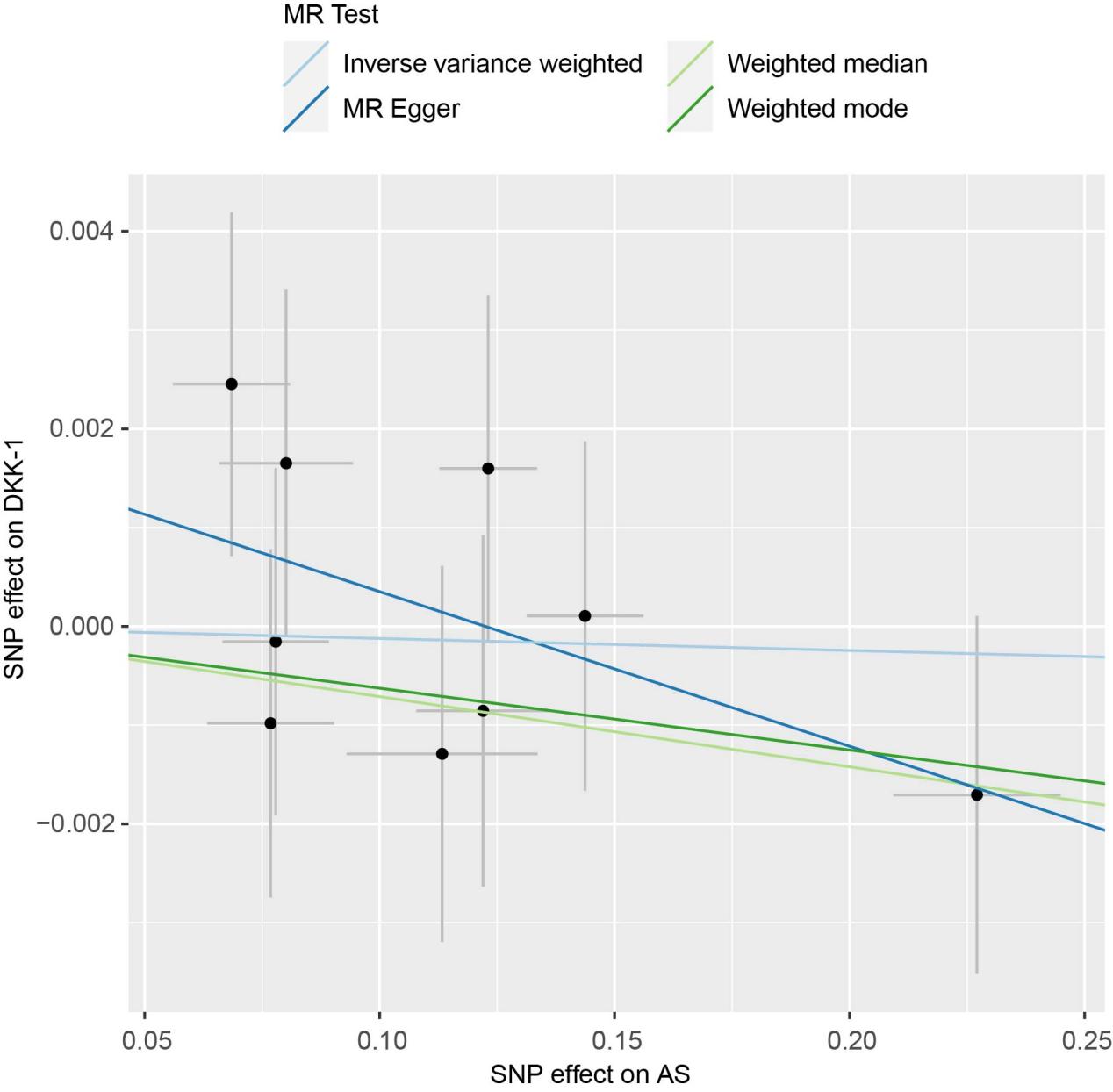


Supplementary Figure 10. Funnel plot of the causality between DKK-1 and AS


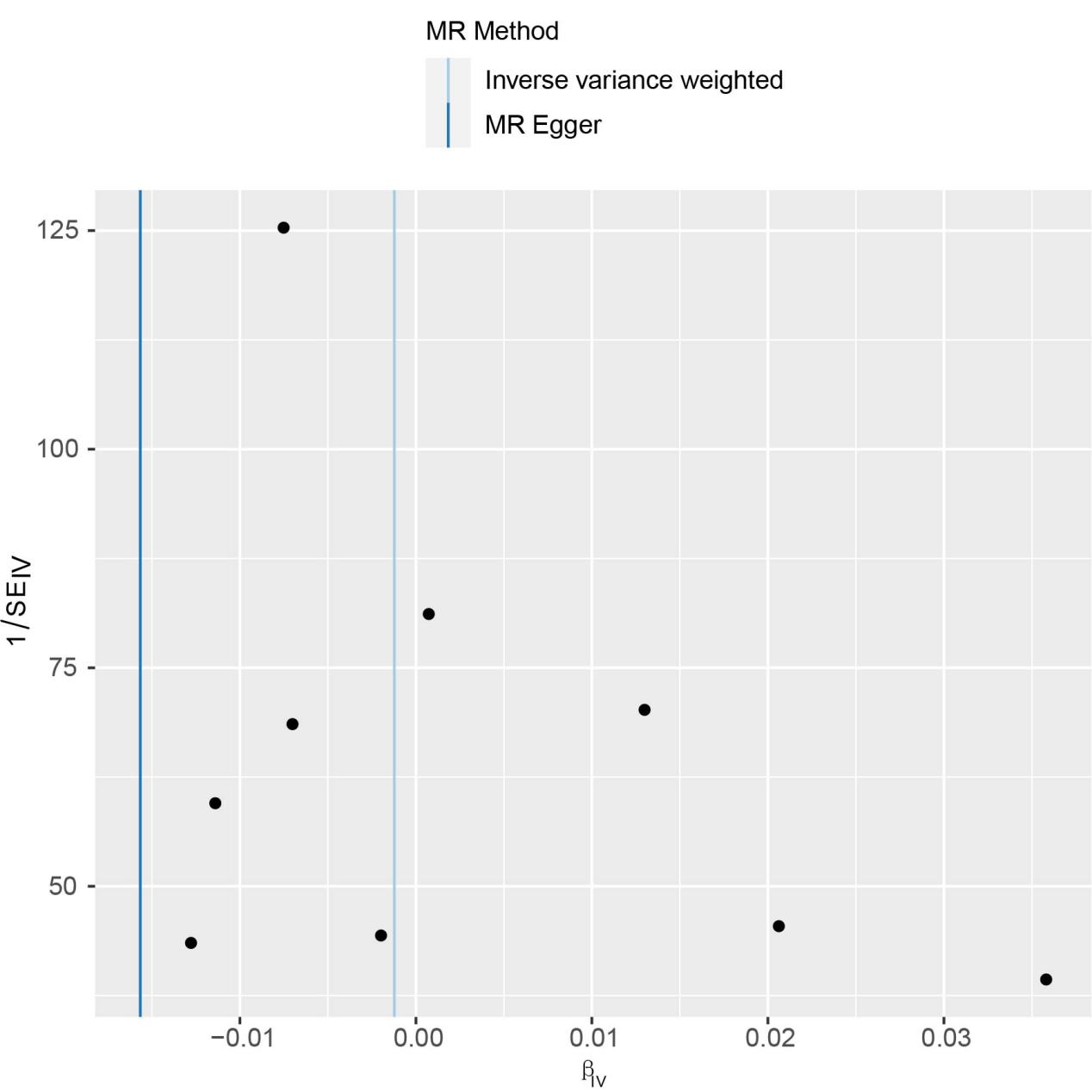


Supplementary Figure 11. Forest plot of the causality between DKK-1 and AS


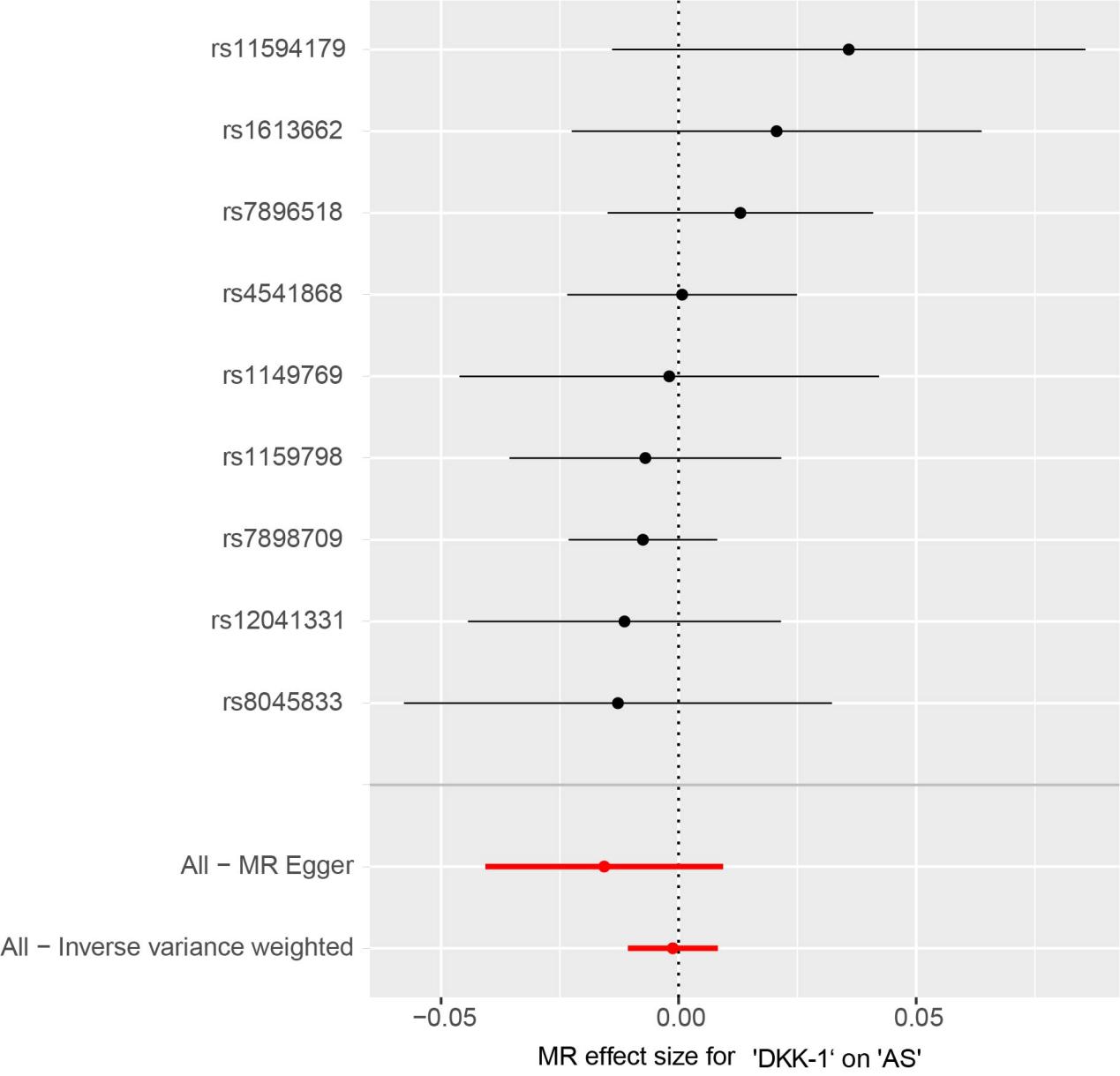

Supplement: Supplementary file 1 [file DataSheet_1.docx]
