# Supplementary material for: Serum DKK-1 level in ankylosing spondylitis: insights from meta-analysis and Mendelian randomization
Source: Front Immunol. 2023 Jul 12;14:1193357. doi: 10.3389/fimmu.2023.1193357 (PMC10368999; doi:10.3389/fimmu.2023.1193357)
Supplement: Supplementary file 2 [file DataSheet_2.docx]

Supplementary Table 1. Quality evaluation of the all included literature

|  |  | Selection | | | |  | Comparablity |  | Exposure | | |  |  |
| --- | --- | --- | --- | --- | --- | --- | --- | --- | --- | --- | --- | --- | --- |
|  |  | Is the Case  Definition Adequate? | Representativeness of the Cases | Selection of Controls | Definition of Controls |  |  |  | Ascertainment  of Exposure | Same method of  ascertainment for cases and controls | Non-Response Rate |  |  |
| Daoussis, 2010 |  | ✬ |  |  | ✬ |  | ✬✬ |  | ✬ | ✬ | ✬ |  | 7 |
| Wang, 2011 |  | ✬ |  |  | ✬ |  |  |  | ✬ | ✬ | ✬ |  | 5 |
| Shan, 2011 |  | ✬ |  |  | ✬ |  | ✬✬ |  | ✬ | ✬ | ✬ |  | 5 |
| Sui, 2011 |  | ✬ |  |  | ✬ |  | ✬✬ |  | ✬ | ✬ | ✬ |  | 7 |
| Elshishtawy, 2012 |  | ✬ |  |  | ✬ |  | ✬✬ |  | ✬ | ✬ | ✬ |  | 7 |
| Kim, 2012 |  | ✬ |  |  | ✬ |  | ✬✬ |  | ✬ | ✬ | ✬ |  | 7 |
| Kwon, 2012 |  | ✬ |  |  | ✬ |  | ✬✬ |  | ✬ | ✬ | ✬ |  | 7 |
| Liu, 2012 |  | ✬ |  |  | ✬ |  |  |  | ✬ | ✬ | ✬ |  | 5 |
| Korkosz, 2013 |  | ✬ |  |  | ✬ |  |  |  | ✬ | ✬ | ✬ |  | 5 |
| Tuylu, 2014 |  | ✬ |  |  | ✬ |  | ✬✬ |  | ✬ | ✬ | ✬ |  | 7 |
| Ustun, 2014 |  | ✬ |  |  | ✬ |  | ✬✬ |  | ✬ | ✬ | ✬ |  | 7 |
| Zhang, 2014 |  | ✬ |  |  | ✬ |  | ✬✬ |  | ✬ | ✬ | ✬ |  | 7 |
| Kong, 2014 |  | ✬ |  |  | ✬ |  | ✬✬ |  | ✬ | ✬ | ✬ |  | 7 |
| Zhou, 2014 |  | ✬ |  |  | ✬ |  | ✬✬ |  | ✬ | ✬ | ✬ |  | 7 |
| Xie, 2015 |  | ✬ |  |  | ✬ |  | ✬✬ |  | ✬ | ✬ | ✬ |  | 7 |
| Xie, 2015 |  | ✬ |  |  | ✬ |  | ✬✬ |  | ✬ | ✬ | ✬ |  | 7 |
| Cui, 2015 |  | ✬ |  |  | ✬ |  | ✬✬ |  | ✬ | ✬ | ✬ |  | 7 |
| Huang, 2016 |  | ✬ |  |  | ✬ |  | ✬✬ |  | ✬ | ✬ | ✬ |  | 7 |
| Su, 2016 |  | ✬ |  |  | ✬ |  | ✬✬ |  | ✬ | ✬ | ✬ |  | 6 |
| Rossini, 2016 |  | ✬ | ✬ |  | ✬ |  | ✬✬ |  | ✬ | ✬ | ✬ |  | 8 |
| Sakellariou, 2017 |  | ✬ |  |  | ✬ |  | ✬✬ |  | ✬ | ✬ | ✬ |  | 7 |
| Niu, 2017 |  | ✬ |  |  | ✬ |  | ✬✬ |  | ✬ | ✬ | ✬ |  | 7 |
| Bai, 2017 |  | ✬ |  |  | ✬ |  | ✬✬ |  | ✬ | ✬ | ✬ |  | 7 |
| Park, 2017 |  | ✬ | ✬ |  | ✬ |  | ✬✬ |  | ✬ | ✬ | ✬ |  | 8 |
| Jadon, 2017 |  | ✬ | ✬ |  | ✬ |  |  |  | ✬ | ✬ | ✬ |  | 6 |
| Liao, 2018 |  | ✬ |  |  | ✬ |  | ✬✬ |  | ✬ | ✬ | ✬ |  | 7 |
| Zhang, 2018 |  | ✬ |  |  | ✬ |  | ✬✬ |  | ✬ | ✬ | ✬ |  | 7 |
| Sun, 2018 |  | ✬ |  |  | ✬ |  |  |  | ✬ | ✬ | ✬ |  | 5 |
| Sohn, 2018 |  | ✬ | ✬ |  | ✬ |  | ✬✬ |  | ✬ | ✬ | ✬ |  | 8 |
| Solmaz, 2018 |  | ✬ | ✬ |  | ✬ |  | ✬✬ |  | ✬ | ✬ | ✬ |  | 8 |
| Sun, 2019 |  | ✬ |  |  | ✬ |  | ✬✬ |  | ✬ | ✬ | ✬ |  | 7 |
| Wang, 2019 |  | ✬ |  |  | ✬ |  |  |  | ✬ | ✬ | ✬ |  | 5 |
| Liu, 2019 |  | ✬ |  |  | ✬ |  | ✬✬ |  | ✬ | ✬ | ✬ |  | 7 |
| Jiao, 2019 |  | ✬ |  |  | ✬ |  | ✬✬ |  | ✬ | ✬ | ✬ |  | 7 |
| Liu, 2020 |  | ✬ |  |  | ✬ |  | ✬✬ |  | ✬ | ✬ | ✬ |  | 7 |
| Pei, 2020 |  | ✬ |  |  | ✬ |  | ✬✬ |  | ✬ | ✬ | ✬ |  | 7 |
| Wang, 2020 |  | ✬ |  |  | ✬ |  | ✬✬ |  | ✬ | ✬ | ✬ |  | 7 |
| Hu, 2021 |  | ✬ |  |  | ✬ |  | ✬✬ |  | ✬ | ✬ | ✬ |  | 7 |
| Hu, 2021 |  | ✬ |  |  | ✬ |  | ✬✬ |  | ✬ | ✬ | ✬ |  | 7 |
| Jo, 2021 |  | ✬ |  |  | ✬ |  | ✬✬ |  | ✬ | ✬ | ✬ |  | 7 |

Supplementary Table 2. MR results of causality between DKK-1 and AS risk

| **Exposure** | **Outcome** | **Methods** | **Nsnp** | **Beta** | **SE** | ***P* value** | **OR (95% CI)** | **Horizontal pleiotropy** | | |  | **Heterogeneity** | | ***F* statistic** |
| --- | --- | --- | --- | --- | --- | --- | --- | --- | --- | --- | --- | --- | --- | --- |
|  |  |  |  |  |  |  |  | **MR-Egger regression** | | | **MR-PRESSO** | **Cochran’s Q** | ***P* value** |  |
|  |  |  |  |  |  |  |  | **Egger intercept** | **SE** | ***P* value** | **Global test P value** |  |  |  |
| DKK-1 | Ankylosing spondylitis | Weighted median | 9 | -0.0071 | 0.0063 | 0.26 | 0.993(0.981, 1.005) | 0.0019 | 0.0016 | 0.2612 | 0.70 | 5.53 | 0.70 | 76.0 |
|  |  | MR Egger | 9 | -0.0157 | 0.0128 | 0.26 | 0.984(0.960, 1.009) |  |  |  |  |  |  |  |
|  |  | Weighted mode | 9 | -0.0063 | 0.0068 | 0.39 | 0.994(0.981, 1.007) |  |  |  |  |  |  |  |
|  |  | Inverse variance weighted | 9 | -0.0012 | 0.0048 | 0.80 | 0.999(0.989, 1.008) |  |  |  |  |  |  |  |

Supplementary Table 3. MR results of causality between DKK-1 and confounding factors

| **Exposure** | **Outcome** | **Nsnp** | **IVW *OR* (95% *CI*)** | ***P* value** |
| --- | --- | --- | --- | --- |
| DKK-1 | Diabetes | 9 | 1.000(0.997, 1.003) | 0.97 |
| DKK-1 | Hypertension | 9 | 1.001(0.993, 1.021) | 0.34 |

Supplementary Table 4. MR results of causality between confounding factors and AS

| **Exposure** | **Outcome** | **Nsnp** | **IVW *OR* (95% *CI*)** | ***P* value** |
| --- | --- | --- | --- | --- |
| Diabetes | Ankylosing spondylitis | 69 | 1.048(0.952, 1.155) | 0.34 |
| Hypertension | Ankylosing spondylitis | 219 | 0.999(0.974, 1.024) | 0.92 |
